# Weekend handover: Improving patient safety during weekend services

**DOI:** 10.1016/j.amsu.2020.06.005

**Published:** 2020-06-09

**Authors:** Rajvi Nagrecha, Jaideep Singh Rait, Kim McNairn

**Affiliations:** a Medway Maritime Hospital, Medway NHS Trust, Windmill Road, Gillingham, ME7 5NY, Kent, UK; b William Harvey Hospital, East Kent NHS Trust, Kennington Rd, Willesborough, TN24 0LZ, Ashford, UK

**Keywords:** Weekend handover, Patient safety, Stress

## Abstract

Clinical Handover has been identified as one of the most high-risk processes within medicine. Inadequate handover is a significant cause of avoidable adverse events across many hospitals. A likert-survey of the weekend handover system at a district general hospital demonstrated significant dissatisfaction amongst junior doctors. Intending to improve patient safety and reduce stress for on-call junior doctors, a weekend handover proforma was compiled according to the Royal College of Physicians and Surgeons guidelines. The proforma was trialed on six medical wards for six months with a before and after questionnaire being sent to doctors on the wards involved to determine the proforma’s merits on a scale of 1 (least effective) to 10 (most effective). Reports subsequent to implementation demonstrated a 67% increase ease of identifying outstanding weekend jobs. 57% of doctors reported better understanding of their patient’s diagnosis and management plan and 53% stated it was easier to identify the patients that required regular medical review over the weekend. Results also highlighted a 55% reported an increase in safety of weekend handovers (p<0.01). A closed loop audit of handover practice through the use of a standardised proforma showed improved quality, detail and consistency of handovers. The reduction in stress for junior doctors managing unknown patients with a clear concise plan, directed by a senior from the parent team during the week, has improved patient safety and doctor satisfaction.

## Highlights

• Weekend handovers are a valuable tool to increase patient safety.
• Weekend handovers can reduce stress levels in junior doctors.
• Weekend handovers have been shown to reduce the financial and operational burden.

## 1 The context

Handover is defined as the system by which the responsibility for immediate and ongoing care is transferred between healthcare professionals [1]. The General Medical Council's Good Medical Practice states that achieving an effective handover is the duty of every doctor [2].

The NHS has adhered to the European Working Time Directive since 1998. Doctors are now limited to working a maximum of 13 h per shift, which means that they are no longer able to provide continuous care for their patients [3]. In addition, the implementation of the junior doctor's contract has seen a change in working patterns. These changes create a significant need for effective handover to ensure continuity of care and by proxy patient safety.

## 2 The problem

The Royal College of Physicians has identified ‘handover, particularly of temporary ‘on-call’ responsibility, as a point at which errors are likely to occur’ [4]. Poor handover between clinicians can result in missed or delayed diagnosis, repeated investigations, incorrect treatment and poor communication with the patient. It is well documented that there is a higher mortality and morbidity of patients on weekends compared to weekdays in NHS hospitals [5]. Poor handovers between the patient's regular team and on-call doctors is a significant contributing factor to this [[6], [7], [8]].

## 3 Key measures of improvement

The Royal College of Surgeons of England issued a guidance from the Working Time Directive working party on safe handover [9]. It highlights that in order for a handover to be safe it should include the following information:

- Patient name and age 
- Date of admission
- Location (ward and bed)
- Responsible consultant
- Current diagnosis
- Results of significant or pending investigations
- Urgency/frequency of review required
- Management plan, including “what if … "
- Any outstanding tasks

Unfortunately, studies have shown that one or more of these components are routinely left out of patient handovers [10,11].

We believe that a Weekend handover proforma, completed for every patient during the Friday ward round, will benefit patient safety. The proforma will contain a summary of the patients background as well as the weekend plan. We hypothesize that this will improve continuity of care for patients, save time and reduce stress for on-call doctors when dealing with unfamiliar patients. Standardized handover proformas have been shown to improve the efficiency of information transfer and reduce adverse events [[12], [13], [14]].

## 4 Institutional setting

In the district general hospital (DGH) studied, junior doctors expressed dissatisfaction with the pre-existing weekend handover system. Over the weekend, two newly qualified junior doctors (house officers) and one medical registrar are responsible for looking after adult patients on all medical wards.

Every Friday at 5pm, the on-call house officers attend a handover meeting, where a patient's regular doctor verbally discusses and ‘hands-over’ a handwritten piece of paper that briefly outlines the weekend plan for the patient. This could range from a medical review for the patient, any investigations to complete or medication to prescribe over the weekend. These handover sheets are not standardized and vary greatly in structure and content. They frequently contain insufficient information about the patient's diagnosis and background and often lack clear instructions for the on-call doctor to follow.

In addition to the patients handed over, the on-call junior doctors also have to manage acutely unwell patients that deteriorate unexpectedly over the weekend. The DGH studied has a handwritten paper-based system for clinical notes, prescriptions and investigation requests. This means patients often have extensive medical notes that are unordered and difficult to navigate. As a result, on-call doctors treating patients for the first time, frequently make clinical decisions without having all the information to hand; resulting in repeated investigations, poor prioritization and sub-optimal care over weekends.

## 5 Process of gathering information about the problem

The study was split into three phases:

1. Baseline survey to assess the level of satisfaction with the pre-existing weekend handover system
2. Design and implementation of a weekend handover proforma
3. Post intervention survey to assess the level of satisfaction amongst doctors following introduction of the weekend handover proforma

A baseline electronic survey (Appendix A) was disseminated to all doctors involved in weekend handovers (house officers, senior house officers and registrars). The survey consisted of Likert scale and qualitative questions to assess the level of satisfaction with the pre-existing weekend handover system and identify areas for improvement. Surveys were answered anonymously. The study was compliant with SQUIRE 2.0 criteria.

**Fig. 1:**
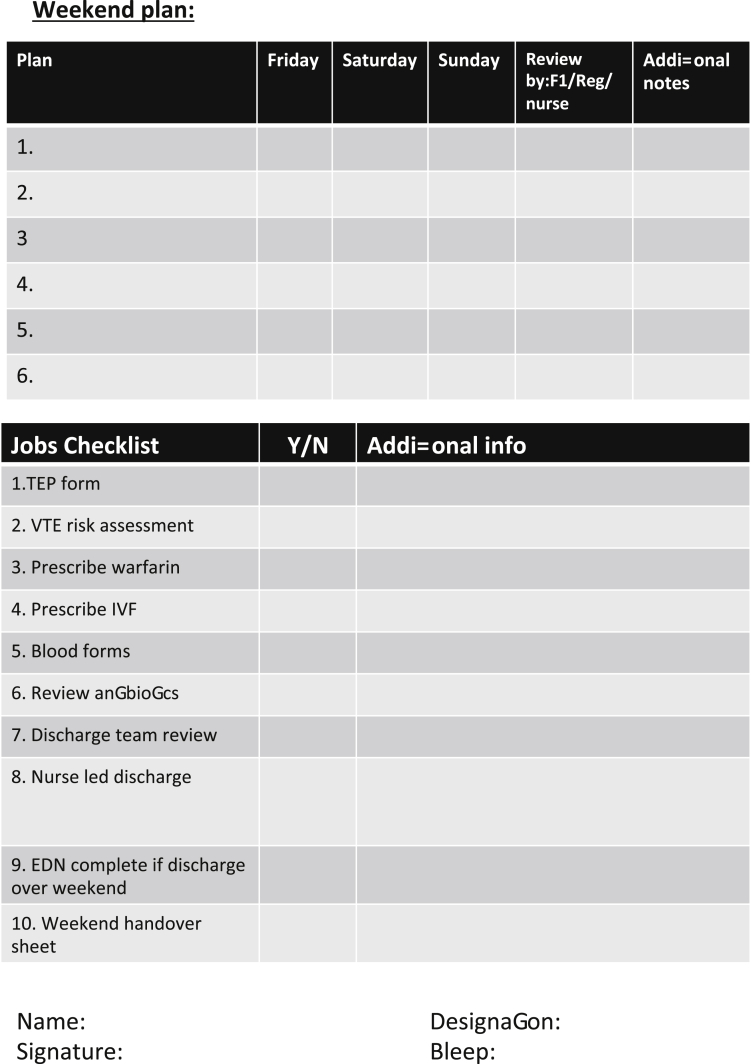
Weekend Handover proforma. Double sided A4 document.

We used the standards from the Royal College of Physicians [15], the guidance from the Royal College of Surgeons [16] as well as the feedback from our baseline questionnaire (appendix B) to create a weekend handover proforma (Fig. 1).

The proforma was designed to be completed during the Friday morning ward round by the patients' regular medical team. The idea being that every patient should have a handover sheet in their notes, regardless of whether then need to be verbally handed over to the on-call team. It will replace the regular Friday ward round documentation and will include an up-to-date summary of the patient's diagnosis, weekend plan and a jobs checklist to be completed ahead of the weekend.

## 6 Analysis and interpretation

30 doctors completed the electronic survey at baseline. 19 doctors completed the survey after the weekend handover proforma had been in use for 6 months.

The pre-intervention survey suggests that doctors were dissatisfied with the pre-existing weekend handover system. Most felt that it was unsafe (83%), difficult and time consuming to determine the diagnosis and management plan from the medical notes (90%) and the majority failed to identify any outstanding jobs and investigations that needed completing over the weekend (70%). 80% of respondents felt that tasks that should have be carried out by the patient's regular team did not get completed prior to the weekend. Overall, 51% of respondents felt that a weekend handover proforma would reduce adverse events and mortality. However, only 33% of doctors believed that the proformas would be completed in sufficient detail during Friday morning ward rounds.

The results from the post-intervention survey showed that doctors valued the new weekend handover proforma (Table 1) and all results showed statistical significance with a p value of <0.01. A paired T-test was used to calculate p values for pre and post intervention scoring. The weighted mean subjective assessment of the safety of weekend handover went up by 55% from 6.67 out of 10 to 3 out of 10 (95% CI p < 0.01). This was assessed by asking doctors how safe they felt weekend handover was on a scale of 0–10, with 0 being very safe and 10 not safe at all. In addition, 53% of respondents felt that the weekend handover proforma had reduced mortality and adverse events on weekends and improved patient care.

**Table 1:** Results from post-intervention survey.

Survey Question	Pre-intervention Weighted average	Post-intervention Weighted average	% change	P-Value
How safe do you feel the weekend handover is at present?	6.67	3.00	55.0%	<0.01
Very Safe (0) to Very Unsafe (10)				
Over the weekend, how **difficult** is it to understand a patient's diagnosis, management plan and ongoing significant issues by reading the medical notes?	6.70	2.89	56.9%	<0.01
Very Easy (0) to Very Difficult (10)				
Over the weekend how **time consuming** is it to understand a patient's diagnosis, management plan and ongoing significant issues by reading the medical notes?	8.03	2.79	65.3%	<0.01
Not Time Consuming (0) to Very Time Consuming (10)				
How difficult is it to identify outstanding jobs and investigations to chase for a patient over the weekend	7.40	2.42	67.3%	<0.01
Very Easy (0) to Very Difficult (10)				
How difficult is it to identify the patients that will need a regular medical review by a certain grade of doctor over the weekend?	2.67	1.26	52.8%	<0.01
Very Easy (0) to Very Difficult (4)				
How satisfied are you that tasks that should be carried out by the patient's regular team get completed prior to the weekend?	3.07	1.58	48.5%	<0.01
Very Satisfied (0) to Very Dissatisfied (4)				

The most significant improvement (67%) following our intervention was noted to be in ease of identifying outstanding jobs and investigations to chase for a patient over the weekend (95% CI p < 0.01). Similarly, doctors found it on average, 57% easier to understand a patient's diagnosis, management plan and ongoing significant issues after looking at the medical notes (95% CI p < 0.01). They also found it on average, 53% easier to identify the patients that will need a regular medical review by a certain grade of doctor over the weekend (95% CI p < 0.01).

## 7 Strategy for change

The handover proforma was trialed on six medical wards for six months. To allow staff to familiarize themselves with the proforma, a trust wide email was sent to all healthcare professionals informing them of the new handover process. Additionally, the proforma was presented at the local patient safety conference prior to being introduced, as well as to junior medical professionals at mandatory teaching sessions. The sheet was made pink so that it could easily be found in the notes. The ward clerks inserted the handover sheet in each of the patient notes on Thursday evenings in preparation for the Friday morning ward rounds.

Following implementation of the proforma for six months, the electronic survey was sent out and disseminated to doctors involved with weekend handovers (house officers, senior house officers and registrars) (appendix a).

The hypothesis was to compare the difference in perceived patient safety and levels of satisfaction amongst doctors before and after implementation of the weekend handover proforma. This was assessed using a *t*-test (with unequal variances), a p-value < 0.05 was considered statistically significant.

## 8 Effects of change

The post-intervention questionnaire revealed that on average, doctors were 49% more satisfied that tasks that should be carried out by the patient's regular team (e.g. warfarin dosing, treatment escalation forms, blood forms, antibiotic reviews, discharge notifications and drug charts) were being completed prior to the weekend (95% CI p < 0.01).

Only 21% of respondents felt that junior doctors were consistently completing the weekend handover proforma in enough detail every Friday prior to the weekend.

Free text comments (Table 2 below) post-intervention highlighted that doctors would like the proforma to be used more widely within the hospital. Doctors also felt that it still required more buy-in from juniors and seniors alike, and they felt an electronic formal would be useful.

**Table 2:** Feedback from post intervention survey.

Respondents feedback	Free text comments from post-intervention survey	How to improve weekend handover proforma
	‘First step in weekend handover improvement’	‘Introduced to the surgical department’
	‘Those that are filled out well make it much easier when working the weekends’	‘Switch to electronic format, as it will make it easier to identify patients that need reviewing/jobs chasing’
	‘Overall really helpful, especially when on call’	‘Bigger box for ‘examination section’
	‘Certainly, helps with a brief overview of the patient diagnosis and jobs needed’.	‘More buy in from juniors and seniors and usage hospital wide’
	‘The weekend handover sheets are definitely useful. But I have noticed that not all jobs get done on weekends. This is likely because of not enough junior doctors.’	

## 9 Lessons learnt

This study highlighted significant inefficiencies and safety concerns with the pre-existing weekend handover system. The results suggest that a weekend handover proforma improves patient safety over weekends and reduces stress and workload for doctors. The effectiveness of similar handover proformas have been reported in the past.

When doctors are working on-call over weekends, they are required to treat a large number of unfamiliar patients under time-pressure. In doing so, the need for an easily accessible, up-to-date summary of the patient's diagnosis and background is key. Thi ensures that doctors are fully informed before making clinical decisions and prioritizing jobs; in turn improving patient safety. Following introduction of the weekend handover proforma, doctors reported finding it on average, over 50% easier and less time-consuming to understand a patient's diagnosis, management plan and ongoing significant issues by reading the medical notes. In line with this, mean subjective assessment of patient safety went up by 55%. Additionally, the majority of doctors (53%) felt that the new handover proforma had reduced mortality and adverse events on weekends. Furthermore, improved handover will mean that patients do not have to answer the same questions repeatedly; increasing their perception of a professional service.

A succinct summary of the patient's diagnosis, background and important completed or pending investigations will also help to reduce the number of repeated investigations. One of the main reasons for unnecessary repeated investigations is due to clinicians lacking awareness about previous requests for the same test [17]^.^ Ensuring this information is readily available to clinicians is the only way to prevent repeated and unnecessary investigations, helping trusts to save money.

In addition, the weekend handover proforma ensures that the regular team documents a clear plan for their patients ahead of the weekend; improving continuity of care and helping junior on-call doctors to feel more supported. Post-intervention doctors felt that it was, on average 67% easier to identify outstanding jobs and investigations to chase for a patient over the weekend. Nurses working on weekends have also stated that they have found the documented weekend plan useful. They reported improvement in communication with the patient and it means they can directly chase certain jobs, such as doctor's review or incomplete investigations. The clearer documentation also increases accountability; important given the increasingly litigious culture within healthcare [18].

The weekend handover proforma has also reduced stress and workload for on-call doctors as it acts as an aide-memoire for the patient's regular doctors to complete routine tasks ahead of the weekend. Results from the post-intervention questionnaire showed a 61% increase in satisfaction amongst doctors when tasks that required by a patient's regular team are being completed prior to the weekend.

At a time when the NHS is facing significant bed-shortages, the handover proforma has helped to ensure safe and timely discharges over weekends. The proforma highlights which patients can be discharged over the weekend, either by a nurse if they meet certain specified criteria, or following review by the discharge team. There is also a prompt on the proforma for the regular team to ensure that the patients electronic discharge notification is completed ahead of the weekend which will save time and inefficiencies surrounding delayed discharges.

### 9.1 Limitations and lessons

This study was limited by the small sample size and restriction to only medical based wards. Whilst this study demonstrated an improvement in the transfer of information between regular and on-call doctors over the weekend, we did not assess the direct impact that the intervention has on patient outcomes or staff productivity, which is ultimately the goal with any healthcare intervention. Further audits are needed to assess the direct impact on patient outcomes and staff productivity.

In the future we are planning to introduce the weekend handover proforma to surgical wards. We aim to reassess the proforma to evaluate its usage and uptake. We are continuing to raise awareness of the proforma among doctors and there has been a teaching session about the weekend handover proforma at the induction of all new doctors joining the trust. There are of-course other aspects of the weekend handover system that were not addressed, including the handover meeting process, understaffing and doctors rota structure, which are beyond the scope of this study. There are plans to assess and develop these factors in future studies.

In the future, paper records are likely to be replaced by a more efficient electronic system. No doubt an electronic system will make it easier for doctors to access patient records, prioritize jobs and save time by reducing paper work. However, this system would require significant investment in software and hardware will take time to implement. In the mean time a better, inexpensive approach is needed to aid the current process of medical handovers and reduce patient morbidity and mortality.

### 9.2 Conclusion

This study has demonstrated that the weekend handover proforma has improved the quality of weekend handovers in Medicine and has been a significant step in improving patient safety over weekends. Additionally, it has reduced stress for junior doctors by ensuring that regular teams carry out routine jobs for patients ahead of the weekend. The changes have also reduced the financial and operational burden for the hospital by ensuring timely discharges and reducing the number of unnecessary investigations on weekends.

## Ethical approval

Ethical approval not sought as no patient identifiable data was used.

## Funding

No funding.

## Author contributions

Rajvi Nagrecha: data collection, analysis and writing the paper. Jaideep Rait: study concept and design, writing the paper and editing the paper. Kim McNairn: data collection and editing the paper.

## Registration of research studies

Researchregistry5670.

## Guarantor

Jaideep Singh Rait.

## Consent

No patient identifiable data, so no consent was sought.

## Provenance and peer review

Not commissioned, externally peer reviewed.

## Declaration of competing interest

No conflicts of interest.
