# Supplementary material for: Weekend handover: Improving patient safety during weekend services
Source: Ann Med Surg (Lond). 2020 Jun 9;56:77–81. doi: 10.1016/j.amsu.2020.06.005 (PMC7322181; doi:10.1016/j.amsu.2020.06.005)
Supplement: Multimedia component 1 [file mmc1.docx]

Appendix A:

Questionnaire (used for both pre and post intervention to assess response.

<https://www.surveymonkey.co.uk/r/HJ7PXR8>​

It is well documented that there is a higher incidence of mortality and adverse events among inpatients in UK hospitals on weekends compared to weekdays. Weekends can be extremely stressful for doctors; due to poor staffing levels, we have to deal with a large number of unfamiliar patients on many different wards. Documented investigations and weekend plans are difficult to find, if documented at all and we often have to spend time filtering through patient notes to identify current problems and plans.

 We are implementing a new weekend handover sheet to see if this can benefit patient safety, save time, improve continuity of care for patients and reduce stress for junior doctors when dealing with unfamiliar patients.

We would be grateful if doctors can complete this survey before and after we have trialed our paper weekend handover sheet. The handover sheet will detail the patient’s diagnosis, background and weekend plan. It will be completed by junior members of the team every Friday and attached to patient notes.

Top of Form

**Question Title**

1. How safe do you feel the weekend handover is at present?

| 0 - very safe | 1 | 2 | 3 | 4 | 5 | 6 | 7 | 8 | 9 | 10 - very unsafe |
| --- | --- | --- | --- | --- | --- | --- | --- | --- | --- | --- |
| 0 - very safe | 1 | 2 | 3 | 4 | 5 | 6 | 7 | 8 | 9 | 10 - very unsafe |

**Question Title**

2. When you are working on call over the weekend, how **difficult**is it to understand a patient’s diagnosis, management plan and ongoing significant issues by reading the medical notes?

| 0 - very easy | 1 | 2 | 3 | 4 | 5 | 6 | 7 | 8 | 9 | 10 - very difficult |
| --- | --- | --- | --- | --- | --- | --- | --- | --- | --- | --- |
| 0 - very easy | 1 | 2 | 3 | 4 | 5 | 6 | 7 | 8 | 9 | 10 - very difficult |

**Question Title**

3. When you are working on call over the weekend, how **time consuming** is it to understand a patient’s diagnosis, management plan and ongoing significant issues by reading the medical notes?

| 0- not very time consuming | 1 | 2 | 3 | 4 | 5 | 6 | 7 | 8 | 9 | 10 - very time consuming |
| --- | --- | --- | --- | --- | --- | --- | --- | --- | --- | --- |
| 0- not very time consuming | 1 | 2 | 3 | 4 | 5 | 6 | 7 | 8 | 9 | 10 - very time consuming |

**Question Title**

4. How **difficult** is it to identify **outstanding jobs and investigations** to chase for a patient over the weekend?

| 0 - very easy | 1 | 2 | 3 | 4 | 5 | 6 | 7 | 8 | 9 | 10 - very difficult |
| --- | --- | --- | --- | --- | --- | --- | --- | --- | --- | --- |
| 0 - very easy | 1 | 2 | 3 | 4 | 5 | 6 | 7 | 8 | 9 | 10 - very difficult |

**Question Title**

5. How **difficult** is it to identify the patients that will need a **regular medical review** by a certain grade of doctor over the weekend?

| 0 - very easy | 1 - easy | 2 - neutral | 3 - difficult | 4 - very difficult |
| --- | --- | --- | --- | --- |
| 0 - very easy | 1 - easy | 2 - neutral | 3 - difficult | 4 - very difficult |

**Question Title**

6. At present how **satisfied** are you that **tasks** that should be carried out by the patient’s regular team get completed prior to the weekend?
*(e.g. as warfarin dosing, TEP forms, blood forms, antibiotic reviews, IVF, EDNs and drug charts)*

| 0 - very satisfied | 1 - satisfied | 2 - neutral | 3 - dissatisfied | 4 - very dissatisfied |
| --- | --- | --- | --- | --- |
| 0 - very satisfied | 1 - satisfied | 2 - neutral | 3 - dissatisfied | 4 - very dissatisfied |

**Question Title**

7. Do you believe that a clear handover sheet attached to patients notes every Friday outlining diagnosis, background, examination findings and weekend plan would reduce mortality and adverse events on weekends and improve patient care?

yes

no

maybe

**Question Title**

8. How likely do you think it is that teams will complete this handover sheet every Friday with appropriate detail?

| 0 - extremely likely | 1 - likely | 2 - neutral | 3 - unlikely | 4 - extremely unlikely |
| --- | --- | --- | --- | --- |
| 0 - extremely likely | 1 - likely | 2 - neutral | 3 - unlikely | 4 - extremely unlikely |

**Question Title**

Bottom of Form

Appendix B:

*Blank space question: What would you want from a weekend handover proforma?*

- Up-to-date summary of the patient’s diagnosis, past medical history, recent bloods, current treatment plan, ceiling of care and resuscitation status
- weekend jobs with specific actions
- list of jobs to act as a reminder for regular team to complete them prior to weekend e.g. TEP form, blood forms out

**References:**
